# Prognostic importance of visit-to-visit blood pressure variability for micro- and macrovascular outcomes in patients with type 2 diabetes: The Rio de Janeiro Type 2 Diabetes Cohort Study

**DOI:** 10.1186/s12933-020-01030-7

**Published:** 2020-05-02

**Authors:** Claudia R. L. Cardoso, Nathalie C. Leite, Gil F. Salles

**Affiliations:** Department of Internal Medicine, University Hospital Clementino Fraga Filho, School of Medicine, Universidade Federal do Rio de Janeiro, Rua Croton, 72, Jacarepagua, Rio de Janeiro, RJ CEP 22750-240 Brazil

**Keywords:** Blood pressure variability, Cardiovascular outcomes, Cohort study, Microvascular complications, Mortality, Type 2 diabetes

## Abstract

**Background:**

The prognostic importance of an increased visit-to-visit blood pressure variability (BP-VVV) for the future development of micro- and macrovascular complications in type 2 diabetes has been scarcely investigated and is largely unsettled. We aimed to evaluate it in a prospective long-term follow-up study with 632 individuals with type 2 diabetes.

**Methods:**

BP-VVV parameters (systolic and diastolic standard deviations [SD] and variation coefficients) were measured during the first 24-months. Multivariate Cox analysis, adjusted for risk factors and mean BP levels, examined the associations between BP-VVV and the occurrence of microvascular (retinopathy, microalbuminuria, renal function deterioration, peripheral neuropathy) and macrovascular complications (total cardiovascular events [CVEs], major adverse CVEs [MACE] and cardiovascular and all-cause mortality). Improvement in risk discrimination was assessed by the C-statistic and integrated discrimination improvement (IDI) index.

**Results:**

Over a median follow-up of 11.3 years, 162 patients had a CVE (132 MACE), and 212 patients died (95 from cardiovascular diseases); 153 newly-developed or worsened diabetic retinopathy, 193 achieved the renal composite outcome (121 newly-developed microalbuminuria and 95 deteriorated renal function), and 171 newly-developed or worsened peripheral neuropathy. Systolic BP-VVV was an independent predictor of MACE (hazard ratio: 1.25, 95% CI 1.03–1.51 for a 1-SD increase in 24-month SD), but not of total CVEs, cardiovascular and all-cause mortality, and of any microvascular outcome. However, no BP-VVV parameter significantly improved cardiovascular risk discrimination (increase in C-statistic 0.001, relative IDI 0.9%).

**Conclusions:**

Systolic BP-VVV was an independent predictor of MACE, but it did not improve cardiovascular risk stratification. The goal of anti-hypertensive treatment in patients with type 2 diabetes shall remain in controlling mean BP levels, not on decreasing their visit-to-visit variability.

## Background

Long-term visit-to-visit variability of blood pressure (BP-VVV) has been investigated as a predictor for all-cause mortality and cardiovascular outcomes in several prospective and retrospective studies, including various patient populations [1–11]. Previous studies have shown that BP-VVV is reproducible [12] and that long-term BP-VVV has been associated with cardiovascular outcomes and mortality in most [1–8] but not in all of them [9–11]. A recent meta-analysis observed a wide heterogeneity among the included studies, which precluded precise effects estimations, but showed only modest associations between systolic BP-VVV and all-cause and cardiovascular mortality and cardiovascular disease incidence [13]. In this meta-analysis [13], the relative risk of systolic BP-VVV for prediction of cardiovascular events in individuals with specific risk factors for cardiovascular disease or mortality, such as in diabetic and hypertensive patients, was higher than in the general population. So, prospective studies with specific groups of individuals are still necessary to evaluate the prognostic importance of long-term BP-VVV.

Individuals with type 2 diabetes have a well-known high risk of developing cardiovascular and microvascular diseases and are also prone to increased BP-VVV; potentially because of high arterial stiffness and to cardiovascular autonomic neuropathy, two conditions frequently observed in type 2 diabetes. Indeed, several previous studies evaluated the associations between high BP-VVV and adverse micro- and macrovascular outcomes and mortality in patients with type 2 diabetes [4, 14–27]. However, most of them were retrospective analyses [16, 18–20, 22–25, 27] and focused only on specific outcomes [14–20, 23–27], without a more comprehensive analysis of both micro- and macrovascular outcomes and mortality. A recent meta-analysis [28] suggested that the systolic BP-VVV might be a risk marker for adverse micro- and macrovascular outcomes and all-cause mortality in individuals with type 2 diabetes. However, their results were clearly inconclusive because of few studies included, with very high heterogeneity among them; and probable publication bias for some outcomes, particularly evident for cardiovascular disease [28]. Furthermore, only few previous studies [21, 24, 25] addressed, by specific statistical approaches, whether any BP-VVV parameter, beyond of being a predictive marker of any adverse outcome, was also capable of improving risk discrimination for this outcome, which is an essential step before a potential risk marker could be recommended for clinical use.

Therefore, the objectives of this prospective follow-up study were to investigate if long-term BP-VVV impacted on prognosis for micro- and macrovascular complications development and for all-cause mortality, over and beyond traditional risk factors, including mean BP levels, in a cohort of patients with type 2 diabetes. We also evaluated whether any BP-VVV parameter was capable of improving risk discrimination for any of these adverse outcomes.

## Methods

### Participants and baseline procedures

This prospective study included 682 individuals with type 2 diabetes from the Rio de Janeiro Type 2 Diabetes (RIO-T2D) Cohort Study, enrolled between August 2004 and December 2008 and followed-up until June 2019 in the diabetes outpatient clinic of our tertiary-care University Hospital. All participants gave written informed consent, and the local Ethics Committee had previously approved the study protocol. The characteristics of this cohort, the baseline procedures and the diagnostic definitions have been described previously [29–33]. In summary, inclusion criteria were all adult type 2 diabetic individual up to 80 years old with either any microvascular (retinopathy, nephropathy or neuropathy) or macrovascular (coronary, cerebrovascular or peripheral artery disease) complication, or with at least two other modifiable cardiovascular risk factors (hypertension, dyslipidemia or current smoking). Exclusion criteria were the presence of morbid obesity (body mass index ≥ 40 kg/m^2^), advanced renal failure (serum creatinine > 180 μmol/l or estimated glomerular filtration rate < 30 ml/min/1.73 m^2^) or of any serious concomitant disease limiting life expectancy. Specifically for this analysis, 50 individuals who had any of the outcomes in the first 24-months of follow-up were excluded, totaling 632 individuals evaluated for BP-VVV parameters. All participants were submitted to a standard baseline protocol that included a thorough clinical-laboratory evaluation. Diagnostic criteria for diabetic chronic complications were detailed previously [29–33]. In brief, coronary heart disease was diagnosed by clinical or electrocardiographic criteria, confirmed by positive ischemic stress tests. Cerebrovascular disease was diagnosed by history and physical examination, and peripheral arterial disease by an ankle-brachial index < 0.9. The diagnosis of nephropathy needed at least two albuminuria ≥ 30 mg/24 h or proteinuria ≥ 0.5 g/24 h or confirmed reduction of glomerular filtration rate (eGFR ≤ 60 ml/min/1.73 m^2^, estimated by the CKD-EPI equation, or serum creatinine > 130 μmol/l). Peripheral neuropathy was determined by clinical examination (knee and ankle reflex activities, feet sensation with the Semmes–Weinstein monofilament, vibration with a 128-Hz tuning fork, pinprick and temperature sensations) and neuropathic symptoms were assessed by a standard validated questionnaire [31, 34]. Clinic blood pressure (BP) was measured three times using a digital oscillometric BP monitor (HEM-907XL, Omron Healthcare, Kyoto, Japan) with a suitable sized cuff on two occasions two weeks apart at study entry [29]. The first measure of each visit was discarded and BP considered was the mean between the last two readings of each visit. Arterial hypertension was diagnosed if mean systolic (SBP) ≥ 140 mmHg or diastolic BP (DBP) ≥ 90 mmHg or if anti-hypertensive drugs had been prescribed. Laboratory evaluation included fasting glycemia, glycated hemoglobin (HbA_1c_), serum creatinine and lipids. Albuminuria and proteinuria were evaluated in two non-consecutive sterile 24-h urine collections.

### BP measurements and long-term visit-to-visit BP variability

The patients had at least 3–4 annual clinic BP measurements during follow-up [29]. All BP measurements were performed under the standardized protocol previously described using the same digital oscillometric BP monitor (HEM-907XL), during attended clinical visits in the sitting position and after at least a 5 min rest. BP-VVV was estimated for the first 12- and 24-month periods of follow-up as the standard deviation (SD) and the variation coefficient (VC = [SD/mean]*100%) of all BP measurements obtained during these periods, separately for SBP and DBP.

### Follow-up and outcomes assessment

The patients were followed-up regularly at least 3–4 times a year until June 2019 under standardized treatment. The observation period for each patient was the number of months from the date of the first clinical examination to the date of the last clinical visit until June 2019 or the date of the first endpoint, whichever came first. Fifty-two individuals (7.6%) were lost from follow-up and considered censored observations at the date of their last visit; all other participants had complete follow-up until death or June 2019. The primary outcomes were the occurrence of any new macrovascular or microvascular events, even for those participants who already had any macro- or microvascular complication at baseline (in the case of patients with retinopathy and peripheral neuropathy at baseline, this was considered as worsening microvascular complication). Macrovascular outcomes were total cardiovascular events (CVEs: fatal or non-fatal myocardial infarctions [MIs], sudden cardiac deaths, new-onset heart failure, death from progressive heart failure, any myocardial revascularization procedure, fatal or non-fatal strokes, any aortic or lower limb revascularization procedure, any amputation above the ankle, and deaths from aortic or peripheral arterial disease), major adverse cardiovascular events (MACE: non-fatal MIs and strokes plus cardiovascular deaths), and all-cause and cardiovascular mortalities [29, 30]. Macrovascular and mortality endpoints were ascertained from medical records (most non-fatal and fatal in-hospital events were attended at our hospital), death certificates and interviews with attending physicians and patient families, using a standard questionnaire reviewed by two independent observers. Microvascular outcomes were retinopathy development or worsening [32], renal outcomes [33] (new microalbuminuria development, and renal function deterioration [defined as doubling of serum creatinine or end-stage renal failure needing dialysis or death from renal failure], and a composite of them), and peripheral neuropathy development or worsening [31]. Retinopathy and renal outcomes were evaluated by annual examinations [32, 33], whereas peripheral neuropathy was evaluated on two serial specific examinations performed after a median of 6 and 10 years from the baseline examination [31, 34].

### Statistical analyses

Continuous data were described as means (SD) or as medians (interquartile range [IQR]). For initial exploratory analyses, patients were categorized into tertiles of 24-month SBP variability parameter (because there is still no accepted normal/abnormal cut-off values for BP-VVV) and baseline characteristics compared by ANOVA, Kruskal–Wallis or χ^2^ tests, when appropriate. Kaplan–Meier curves of cumulative endpoints incidence during follow-up, compared by log-rank tests, were used for assessing different incidences of outcomes among the tertile subgroups. For assessing the prognostic value for each macrovascular and microvascular outcome, except for peripheral neuropathy, a time-to-event Cox analysis was undertaken with progressively increasing statistical adjustments for potential confounding variables. Model 1 was only adjusted for age, sex, and the number of BP measurements (to account for the possible influence of different number of BP measurements on BP-VVV); Model 2 was further adjusted for other potential confounders (diabetes duration, body mass index [BMI], smoking status, physical inactivity, arterial hypertension, number of anti-hypertensive drugs in use, presence of each micro- and macrovascular complications at baseline, mean HbA_1c_, HDL- and LDL-cholesterol levels during the same time-period of BP-VVV measurement, and use of insulin, statins and aspirin; and Model 3 was further adjusted for mean SBP and DBP levels during the same period of BP-VVV measurement. The correlation between 24-month SBP-SD and mean SBP was only moderate (r = 0.48) and the simultaneous inclusion of both variables did not cause collinearity, as assessed by variance inflation factors (VIF) of less than 3.0 in all models. These results were presented as hazard ratios (HRs) with their 95% confidence intervals (CIs); to allow comparisons among different BP parameters, their HRs were calculated for standardized increments of 1-SD. For peripheral neuropathy analyses, a multiple logistic regression was used with the same progressively increasing statistical adjustments, except that height (instead of BMI) and the time interval between the baseline and the other 2 neuropathy evaluations were included as adjusting covariates. These results were reported as odds ratios (ORs) with their respective 95% CIs, also estimated for increments of 1-SD in each BP-VVV parameter. The same analyses were performed for patients categorized into tertiles of each BP-VVV parameter, with HRs and ORs calculated for the highest tertile subgroup in relation to the reference lowest tertile subgroup, after adjustments for the same covariates. Separate analyses were performed for each microvascular outcome after excluding those patients with this specific complication at baseline. Also, a separate analysis was performed censoring the follow-up at 10 years. If any of the BP-VVV parameters was demonstrated as a significant predictor of any of the outcomes, then the improvement in risk discrimination of adding this BP-VVV parameter over a standard risk factor model (composed by those covariates in Model 3) for this specific outcome was tested by the C-statistic (analogous to the area under ROC curve applied to time-to-event analysis), compared by the method proposed by DeLong [35], and by the integrated discrimination improvement (IDI) index [36, 37]. The IDI is equivalent to the difference in discrimination slopes between models with and without the new variable and its calculation is based on continuous differences in predicted risk in new and old models in individual cases and controls. Both the absolute and the relative IDI were calculated. The relative IDI, reported as a percentage, facilitates the IDI clinical interpretation, and is defined as the increase in discrimination slope divided by the slope of the standard model including only the traditional cardiovascular risk factors [36, 37]. Interactions between BP-VVV parameters and age, sex, diabetes duration, the presence of micro- and macrovascular complications and glycemic control were tested for all endpoints and whenever there was evidence of interaction (p < 0.10 for interaction term), stratified analyses were performed. Statistics were performed with SPSS version 19.0 (SPSS Inc, Chicago, Il., USA) and R version 3.4.1 (R Foundation for Statistical Computing, Vienna, Austria); and a 2-tailed probability value < 0.05 was considered significant.

## Results

### Baseline characteristics

Six-hundred and thirty-two patients without endpoints during the first 24 months of follow-up were evaluated for macrovascular and mortality outcomes. For microvascular outcomes, 614 patients were evaluated for renal, 524 for retinopathy and 506 for peripheral neuropathy outcomes. Patients had a median of 4 BP measurements during the first 12 months of follow-up (range: 2–6), and a median of 7 BP measurements during the first 24 months of follow-up (range: 4–12). Table 1 outlines the baseline characteristics of all 632 patients and of those divided according to tertiles of 24-month SBP variability. Patients with higher systolic BP-VVV were older, with longer diabetes duration, and had higher prevalences of cerebrovascular and peripheral artery disease and of microvascular complications than those with lower SBP variability. They had higher office and ambulatory BP levels, and a poorer glycemic control, although were using more frequently insulin, and had worse LDL-cholesterol control than those with lower SBP variability. They also had a worse renal function and greater albuminuria values.

**Table 1:** Baseline characteristics and outcomes incidence in all diabetic patients and divided into tertiles of 24-month systolic blood pressure visit-to-visit variability (standard deviation)

Characteristics	All patients (n = 632)	1st-tertile SBP-SD ≤ 11.93 mmHg (n = 211)	2nd-tertile SBP-SD 11.94-17.63 mmHg (n = 215)	3rd-tertile SBP-SD ≥ 17.64 mmHg (n = 206)	*p* value
Age (years)	60.0 (9.5)	57.3 (9.7)	60.5 (9.5)	62.3 (8.6)	< 0.001
Male sex (%)	38.6	38.8	42.7	34.5	0.20
BMI (kg/m^2^)	29.7 (4.9)	29.4 (5.0)	29.5 (4.8)	30.2 (4.7)	0.13
Smoking, current/past (%)	45.0	45.2	48.0	41.8	0.39
Physical activity (%)	22.4	26.6	21.8	19.0	0.15
Diabetes duration (years)	8 (3–15)	7 (2–15)	7 (3–13)	10 (4–18)	0.002
Chronic diabetic complications (%)					
Cerebrovascular disease	9.1	4.9	7.1	14.7	0.001
Coronary artery disease	15.1	12.9	13.3	19.0	0.11
Peripheral artery disease	16.8	8.5	18.2	23.4	< 0.001
Retinopathy	32.4	23.4	32.7	40.7	< 0.001
Nephropathy	31.4	26.9	29.9	37.1	0.057
Peripheral neuropathy	29.2	22.1	27.6	37.7	0.001
Cardiovascular autonomic neuropathy	18.5	17.4	17.8	19.8	0.81
Diabetes treatment (%)					
Metformin	87.7	89.2	87.1	86.6	0.67
Sulfonylureas	43.2	47.3	40.4	41.8	0.30
Insulin	47.8	38.2	52.4	52.6	0.031
Aspirin	89.6	83.6	93.3	93.5	0.010
Dyslipidemia (%)	87.1	85.2	89.3	86.6	0.42
Statins use (%)	77.7	74.3	79.6	78.8	0.38
Arterial hypertension (%)	86.5	77.1	89.3	92.7	< 0.001
Number of anti-hypertensive drugs	3 (1–3)	2 (1–3)	2 (1–3)	3 (2–4)	< 0.001
ACE inhibitors/AR blockers (%)	81.2	69.6	84.9	88.8	< 0.001
Diuretics (%)	62.6	47.8	61.3	78.0	< 0.001
Calcium channel blockers (%)	28.0	21.0	29.3	33.6	0.010
Beta-blockers (%)	46.4	35.3	44.0	59.5	< 0.001
Blood pressures (mmHg)					
Mean 12-month clinic SBP	141 (20)	132 (16)	140 (16)	151 (19)	< 0.001
Mean 12-month clinic DBP	80 (10)	78 (8)	79 (9)	82 (10)	< 0.001
Mean 24-month clinic SBP	141 (18)	132 (15)	140 (15)	150 (22)	< 0.001
Mean 24-month clinic DBP	79 (9)	77 (8)	78 (9)	82 (12)	< 0.001
Ambulatory 24 h SBP	129 (15)	124 (13)	128 (12)	134 (18)	< 0.001
Ambulatory 24 h DBP	74 (10)	73 (8)	73 (9)	75 (12)	0.13
Laboratory variables					
Mean 12-month HbA_1c_ (%)	7.7 (1.6)	7.4 (1.4)	7.8 (1.4)	7.8 (1.6)	0.005
Mean 12-month HbA_1c_ (%) (mmol/mol)	61 (17.5)	57 (15.3)	62 (15.3)	62 (17.5)	
Mean 24-month HbA_1c_ (%)	7.7 (1.4)	7.5 (1.5)	7.8 (1.4)	7.8 (1.4)	0.044
Mean 24-month HbA_1c_ (%) (mmol/mol)	61 (15.3)	58 (13.0)	62 (15.3)	62 (15.3)	
Mean 12-month triacylglycerol (mmol/mol)	1.90 (1.54)	1.80 (1.26)	1.76 (1.08)	2.12 (2.03)	0.058
Mean 24-month triacylglycerol (mmol/mol)	1.90 (1.42)	2.12 (1.48)	1.80 (1.04)	2.01 (1.67)	0.21
Mean 12-month HDL-cholesterol (mmol/mol)	1.13 (0.49)	1.18 (0.63)	1.14 (0.32)	1.11 (0.29)	0.19
Mean 24-month HDL-cholesterol (mmol/mol)	1.14 (0.33)	1.16 (0.39)	1.15 (0.30)	1.12 (0.28)	0.34
Mean 12-month LDL-cholesterol (mmol/mol)	2.79 (0.85)	2.71 (0.81)	2.73 (0.81)	2.90 (0.88)	0.030
Mean 24-month LDL-cholesterol (mmol/mol)	2.72 (0.77)	2.63 (0.71)	2.71 (0.76)	2.81 (0.81)	0.037
Glomerular filtration rate (ml/min/1.73 m^2^)	81 (20)	85 (21)	82 (19)	76 (20)	< 0.001
Albuminuria (mg/24 h)	13 (7–41)	12 (6–26)	13 (7–39)	17 (8–72)	0.003
Macrovascular outcomes^a^					
Total CVEs	162 (2.72)	41 (1.99)	57 (2.73)	64 (3.56)	0.002
Major CVEs	132 (2.17)	32 (1.51)	46 (2.16)	54 (2.95)	0.001
Cardiovascular mortality	95 (1.52)	24 (1.11)	29 (1.32)	42 (2.22)	0.003
All-cause mortality	212 (3.38)	51 (2.36)	67 (3.05)	94 (4.96)	< 0.001
Microvascular outcomes^b^					
Retinopathy (incident/worsening) (n = 524)	153 (5.05)	49 (4.42)	49 (4.60)	55 (6.44)	0.031
Renal composite (n = 614)	193 (3.67)	56 (3.08)	66 (3.61)	71 (4.46)	0.048
Microalbuminuria (incident) (n = 565)	121 (2.53)	36 (2.20)	45 (2.73)	40 (2.67)	0.56
Renal failure (n = 614)	95 (1.61)	27 (1.34)	30 (1.45)	38 (2.14)	0.035
Peripheral neuropathy (incident/worsening) (n = 506)	171 (33.8)	46 (24.7)	69 (39.9)	56 (38.1)	0.004

Values are proportions, and means (standard deviations) or medians (interquartile range)

HbA_1c_, glycated hemoglobin; ACE, angiotensin-converting enzyme; AR, angiotensin II receptor; SBP, systolic blood pressure; DBP, diastolic blood pressure; FG, fasting glycemia; HDL, high-density lipoprotein; LDL, low-density lipoprotein; CVEs, cardiovascular events

^a^Values are absolute numbers (incidence rate per 100 patient-years of follow-up)

^b^Values are absolute numbers (incidence rate per 100 patient-years of follow-up), except for peripheral neuropathy that are absolute numbers (proportions)

### Endpoints occurrence during follow-up

**Fig. 1:**
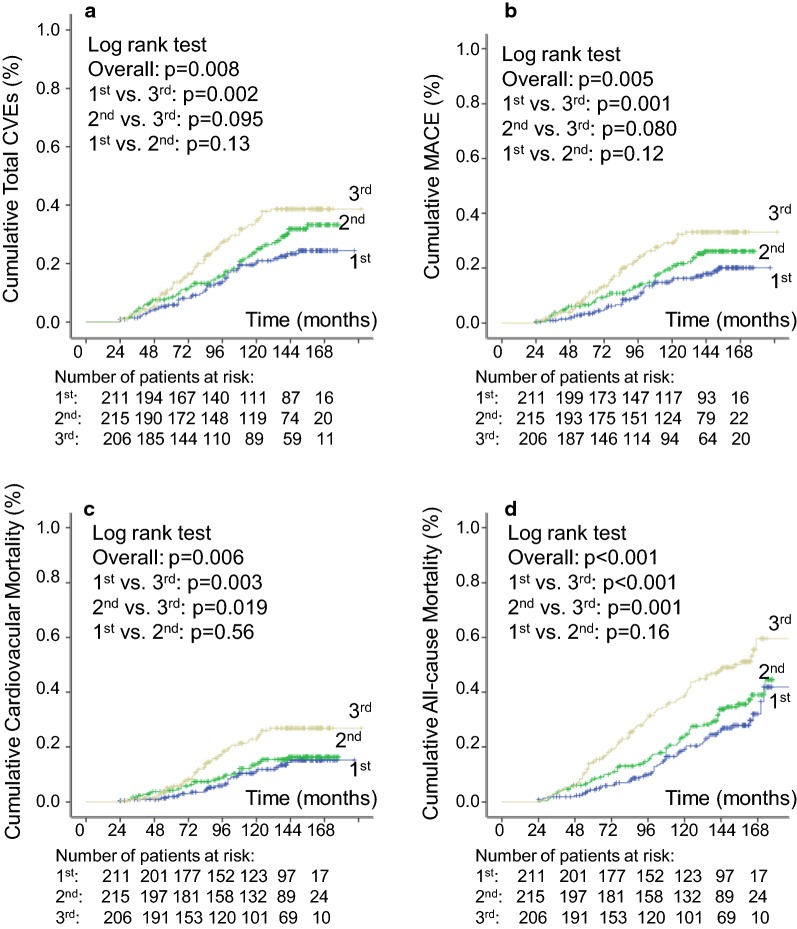
Kaplan–Meier estimation curves of cumulative events incidence during follow-up for total cardiovascular events (CVEs) outcome (**a**), for major adverse cardiovascular events (MACE, **b**), for cardiovascular mortality (**c**), and for all-cause mortality (**d**), in patients divided according to tertiles of 24-month systolic blood pressure visit-to-visit variability (standard deviation)

**Fig. 2:**
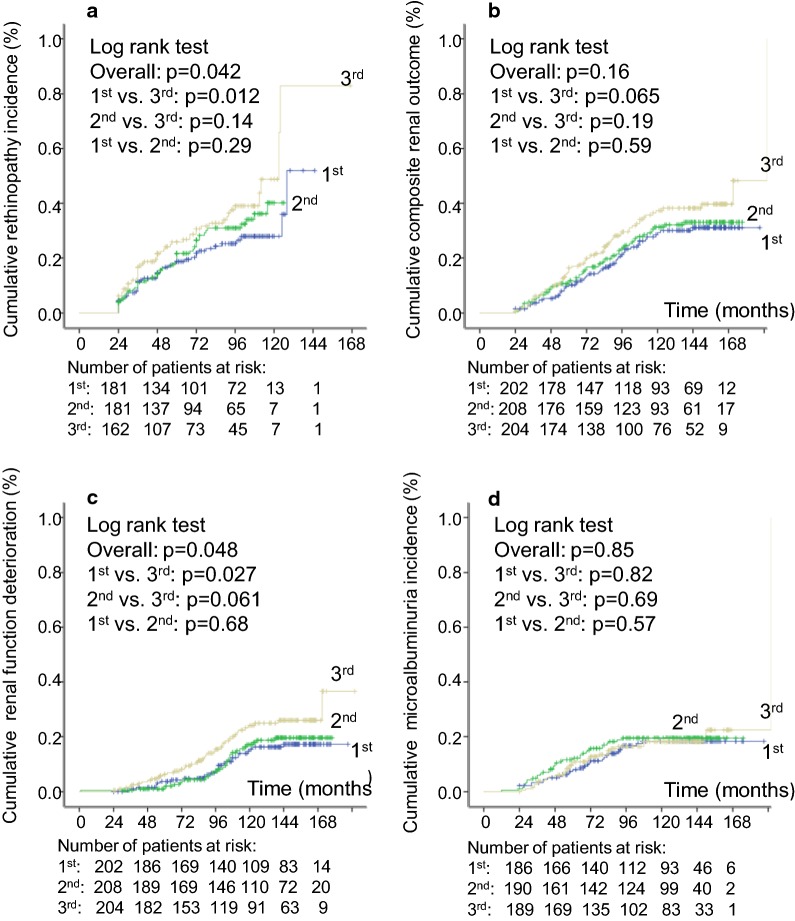
Kaplan–Meier estimation curves of cumulative events incidence during follow-up for the retinopathy outcome (**a**), for the composite renal outcome (**b**), for renal function deterioration (**c**), and for new microalbuminuria development (**d**), in patients divided according to tertiles of 24-month systolic blood pressure visit-to-visit variability (standard deviation)

Over a median follow-up of 11.25 years (IQR: 7.1–13.1 years, maximum 16 years), 162 patients had a CVE after the 2nd year of follow-up (132 MACE); and 212 patients died, 95 from cardiovascular diseases. One-hundred and fifty-three newly-developed or worsened diabetic retinopathy, 193 achieved the renal composite outcome (121 newly-developed microalbuminuria and 95 deteriorated renal function), and 171 newly-developed or worsened peripheral neuropathy. Table 1 shows that patients with higher systolic BP-VVV had a significantly higher incidence of all endpoints, except of microalbuminuria development. Kaplan–Meier curves of cumulative incidence of endpoints (Figs. 1 and 2) show that for most of the endpoints the increased incidence was observed in the highest tertile of SBP variability in relation to the middle and lowest tertile subgroups.

### Risks associated with increased long-term BP variability

Table 2 presents the risks associated with 12-month and 24-month BP-VVV parameters after increasing levels of confounding variables adjustments for macrovascular and mortality outcomes. As a whole, SBP variability parameters were better risk predictors than DBP parameters, and 24-month BP-VVV was better than 12-month BP-VVV parameters. Regarding cardiovascular endpoints, 12-month and 24-month SBP variability parameters, either estimated by the SD or VC, were independent predictors only for MACE occurrence. For total cardiovascular events, all-cause and cardiovascular mortalities, no BP-VVV parameter was an independent risk predictor. Regarding MACE components, SBP variability was associated with a slightly higher risk for strokes (HR: 1.27, 95% CI 0.91–1.77, p = 0.16) than for myocardial infarctions (HR: 1.20; 95% CI 0.90–1.61; p = 0.22), both non-significant after full statistical adjustments because of the smaller number of endpoints (46 MIs and 39 strokes). When categorized into tertiles, the highest tertile subgroup of all BP-VVV parameters had no significant excess risk for any of the macrovascular-mortality outcomes in relation to the lowest tertile subgroup, after full statistical adjustments (Model 3) (data not shown). Also, censoring the follow-up at 10 years, although slightly increased the HRs, it did not materially change any of the results. For example, the HRs for 24-month SBP-SD changed to 1.20 (0.92–1.54) for total CVEs, to 1.23 (0.98–1.52) for MACE, to 1.11 (0.87–1.43) for cardiovascular mortality, and to 1.14 (0.94–1.37) for all-cause mortality after full statistical adjustments (compare with Model 3 in Table 2). Otherwise, no BP variability parameter improved the risk discrimination for MACE occurrence. There was no significant increase in C-statistic: the area under curve of the standard model (with mean SBP) was 0.723 (95% CI 0.674–0.772) and, after adding 24-month SBP-SD or SBP-VC, it was 0.724 (95% CI 0.675–0.773); p = 0.90 for the difference between areas under curve. Also, there was no significant improvement as assessed by the IDI index: absolute IDI 0.001, relative IDI 0.9%, p = 0.57 for both SBP-SD and SBP-VC.

**Table 2:** Results of Cox survival analyses for the excess risks associated with 12-month and 24-month s visit-to-visit blood pressure variability parameters for the occurrence of future macrovascular complications and all-cause mortality

Outcomes	BP variability parameter	12-Month BP variability			24-Month BP variability		
		Model 1 HR (95% CI)	Model 2 HR (95% CI)	Model 3 HR (95% CI)	Model 1 HR (95% CI)	Model 2 HR (95% CI)	Model 3 HR (95% CI)
Total CV events (n = 162)	SBP-SD	1.33 (1.15–1.53)*	1.13 (0.97–1.30)	1.10 (0.95–1.28)	1.37 (1.18–1.58)*	1.17 (0.99–138)	1.12 (0.94-1.33)
	SBP-VC	1.26 (1.10–1.46)^†^	1.10 (0.95–1.26)	1.10 (0.95–1.27)	1.26 (1.10–1.44)^†^	1.12 (0.95–1.31)	1.11 (0.95–1.31)
	DBP-SD	1.15 (1.02–1.31)^‡^	1.06 (0.94–1.20)	1.04 (0.92–1.19)	1.22 (1.07–1.40)^†^	1.11 (0.97–1.27)	1.08 (0.94–1.23)
	DBP-VC	1.12 (0.98–1.27)	1.06 (0.93–1.20)	1.05 (0.92–1.20)	1.22 (1.07–1.40)^†^	1.11 (0.97–1.27)	1.10 (0.96–1.13)
Major CV events (n = 132)	SBP-SD	1.39 (1.19–1.63)*	1.21 (1.03–1.43)^‡^	1.18 (1.00–1.40)^‡^	1.45 (1.23–1.70)*	1.30 (1.09–1.56)^†^	1.25 (1.03–1.51)^‡^
	SBP-VC	1.31 (1.12–1.53)^†^	1.16 (0.99–1.36)	1.17 (0.99–1.37)	1.32 (1.14–1.53)*	1.22 (1.03–1.44)^‡^	1.22 (1.03–1.45)^‡^
	DBP-SD	1.17 (1.03–1.34)^‡^	1.09 (0.96–1.24)	1.07 (0.93–1.22)	1.24 (1.08–1.43)^†^	1.15 (1.00–1.33)^‡^	1.12 (0.97–1.29)
	DBP-VC	1.13 (0.98–1.29)	1.08 (0.94–1.24)	1.07 (0.93–1.23)	1.25 (1.09–1.44)^†^	1.16 (1.00–1.34)^‡^	1.15 (1.00–1.33)^‡^
CV mortality (n = 95)	SBP-SD	1.24 (1.04–1.49)^‡^	1.06 (0.88–1.22)	1.02 (0.84–1.24)	1.29 (1.06–1.55)^‡^	1.08 (0.87–1.33)	1.02 (0.82–1.28)
	SBP-VC	1.13 (0.95–1.35)	1.02 (0.85–1.22)	1.00 (0.84–1.21)	1.15 (0.96–1.39)	1.02 (0.83–1.26)	1.02 (0.83–1.25)
	DBP-SD	1.21 (1.03–1.41)^‡^	1.11 (0.95–1.30)	1.09 (0.92–1.28)	1.24 (1.04–1.48)^‡^	1.12 (0.94–1.33)	1.08 (0.90–1.29)
	DBP-VC	1.14 (0.96–1.34)	1.09 (0.92–1.29)	1.09 (0.92–1.29)	1.20 (1.06–1.35)^‡^	1.11 (0.93–1.32)	1.12 (0.93–1.31)
All–cause mortality (n = 212)	SBP-SD	1.21 (1.07–1.37)^†^	1.06 (0.93–1.20)	1.05 (0.91–1.20)	1.29 (1.13–1.45)*	1.11 (0.96–1.28)	1.07 (0.92–1.25)
	SBP-VC	1.13 (1.00–1.28)^‡^	1.03 (0.91–1.17)	1.03 (0.91–1.17)	1.17 (1.04–1.32)^‡^	1.06 (0.93–1.22)	1.06 (0.92–1.22)
	DBP-SD	1.16 (1.03–1.30)^‡^	1.11 (0.98–1.24)	1.10 (0.98–1.24)	1.24 (1.11–1.40)*	1.16 (1.02–1.31)^‡^	1.14 (0.99–1.29)
	DBP-VC	1.13 (1.00–1.27)^‡^	1.12 (0.99–1.26)	1.11 (0.99–1.26)	1.20 (1.06–1.35)^†^	1.12 (0.99–1.27)	1.12 (0.99–1.27)

Model 1 is adjusted for age and sex, and number of BP measurements

Model 2 is further adjusted for diabetes duration, BMI, smoking status, physical inactivity, arterial hypertension, number of anti-hypertensive drugs in use, presence of micro- and macrovascular complications at baseline, mean HbA_1C_, serum mean HDL- and LDL-cholesterol, and use of insulin, statins and aspirin

Model 3 is further adjusted for mean SBP and DBP

HR, hazard ratio; CI, confidence interval; CV, cardiovascular; SBP-SD, systolic blood pressure standard deviation, SBP-VC, systolic blood pressure variation coefficient; DBP-SD, diastolic blood pressure standard deviation, DBP-VC, diastolic blood pressure variation coefficient

Values are hazard ratios and 95% confidence intervals, estimated for increases of 1-SD in each BP variability parameter.*p < 0.001; ^†^p < 0.01; ^‡^p < 0.05

Regarding microvascular outcomes, no BP-VVV parameter, either analyzed as a continuous variable or categorized into tertiles, predicted any of the outcomes. Although for most microvascular outcomes, except for microalbuminuria development, there were significantly excess risks after age and sex adjustments (Model 1) (with HRs varying from 1.20 for retinopathy to 1.33 for renal function deterioration), after full statistical adjustments including mean BP levels (Model 3), none of them remained significant: for diabetic retinopathy HR = 1.06, 95% CI 0.89–1.26, p = 0.49; for renal function deterioration HR 1.07, 95% CI 0.89–1.28, p = 0.49); and for peripheral neuropathy OR: 1.21, 95% CI 0.93–1.57, p = 0.16 (all estimated for increments of 1-SD in 24-month SBP-SD). Excluding the individuals with pre-existent microvascular complications at baseline did not change any of the results.

There were no evidences of interactions between any of the long-term BP-VVV parameters and age, sex, diabetes duration, presence of diabetic complications at baseline and glycemic control, regarding all outcomes (all p > 0.15 for the interaction terms).

## Discussion

### Main findings

This prospective study, with a median follow-up of 11.3 years, investigated the prognostic importance of BP-VVV for micro- and macrovascular outcomes and for all-cause mortality, beyond traditional risk factors, in a middle-aged type 2 diabetes cohort. First, it demonstrated that SBP-VVV (SD and VC) were independent predictors of MACE, but not of total cardiovascular events, and of cardiovascular and all-cause mortalities. Systolic BP-VVV parameters seemed to be slightly stronger predictors of strokes than of myocardial infarctions. However, none of the systolic BP-VVV parameters were capable of significantly improving risk discrimination for future MACE occurrence. Second, SBP variability parameters were better cardiovascular risk markers than DBP parameters and BP-VVV parameters estimated during the first 2 years of follow-up were better than those estimated during the first year. Finally, no BP-VVV parameter predicted any of the microvascular outcomes.

### Previous studies on BP-VVV

Since the pioneering study of Rothwell and colleagues [1] demonstrating that a large systolic BP-VVV was associated with higher risks of strokes in individuals with previous transient ischemic attacks and of cardiovascular events in hypertensive patients, BP-VVV has been extensively evaluated as an additional predictor of worse cardiovascular outcomes independent of mean BP levels in several clinical settings [2–11, 13], including in patients with type 2 diabetes [4, 14–27], but still with controversial findings. Most of the previous studies in type 2 diabetes were retrospective analyses [16, 18–20, 22–25, 27], and estimated the BP-VVV during the whole follow-up period [15, 18–20, 26] or in the year preceding the outcome [23], which may have provoked reverse causation (i.e., it might be the proximity of the outcome, particularly the fatal ones, that caused the increase in BP-VVV, not the opposite). Moreover, a recent meta-analysis on the prognostic value of long-term BP-VVV in type 2 diabetes [28] was clearly inconclusive because of the few studies included (5–7 depending on the outcome), the high heterogeneity among them (I^2^ statistic between 55% and 93%) and the possibility of publication bias (particularly evident for cardiovascular outcomes). Hence, new studies on this issue are still needed, particularly prospective analyses with comprehensive assessments of both micro- and macrovascular outcomes and mortality, such as the present one.

### BP-VVV and mortality in diabetes

Regarding the importance of BP-VVV for all-cause mortality in type 2 diabetes, some previous studies reported that the systolic BP-VVV may be a risk marker [4, 15, 17, 21, 22]; whereas others reported no value in the whole cohort [14, 24], but only in some subgroup analyses, such as in older individuals [14] or in those not using anti-hypertensive medications [24]. Notably, the Action in Diabetes and Vascular Disease: Preterax and Diamicron Modified Release Controlled Evaluation (ADVANCE) trial reported that the SBP-SD was predictive of all-cause mortality, but the associated risks attenuated with a longer follow-up (HR of 1.29 in the initial 2.4 years of follow-up after the BP-VVV measurement period, and of 1.13 for the extended follow-up of 7.6 years) [4, 21]. Indeed, most of the positive studies had median follow-ups of less than 4 years [17, 22]. Hence, our study with the longest median follow-up of 11.3 years (maximum of 16 years), might have failed to find any prognostic value of BP-VVV for all-cause mortality because of its longest follow-up. However, when we analyzed only the first 10 years of follow-up, although the BP-VVV-related HRs slightly increased, none of them reached statistical significance. This suggests that it was not only the longer follow-up that explained the lack of prognostic value of BP-VVV parameters for mortality, although it may have contributed to it. On the other hand, the fact that the studies with shorter follow-ups did demonstrate a significant predictive value of BP-VVV for all-cause mortality may suggest that reverse causation might have played at least a partial role in determining these associations. Indeed, except for the ADVANCE-ON (extended follow-up) study, the only one positive study with a follow-up longer than 4 years [15] measured the BP-VVV during the entire follow-up period; thus, also prone to reverse causation. This study [15] and the ADVANCE with a shorter follow-up [4] were also the unique ones to show associations between BP-VVV and cardiovascular mortality. All other studies [21, 24], including the ADVANCE with extended follow-up [21], in agreement with our results, did not demonstrate any prognostic importance of BP-VVV for cardiovascular mortality.

### BP-VVV and cardiovascular events in diabetes

We demonstrated that the systolic BP-VVV was an independent predictor only for MACE occurrence, slightly stronger for strokes than for myocardial infarctions. We corroborate with data from the ADVANCE trial [4, 21], from a retrospective Chinese cohort from Hong-Kong [22], and from a retrospective cohort from China [27]. However, only the ADVANCE-ON cohort [21] assessed whether BP-VVV parameters were capable of improving cardiovascular risk discrimination, reporting modest but significant improvements in C-statistic (+0.003) and in relative IDI (4.1%), but without significant improvements when measured by the continuous or categorical Net Reclassification Improvement (NRI) index. We found a nonsignificant minimum improvement in the C-statistic (+0.001) and in IDI (0.9%). In conjunction, these data suggest that the contribution of BP-VVV for improving cardiovascular risk stratification over a standard risk factor model that includes mean BP levels is at most very modest. In this regard, a recent cohort study confirmed the pivotal role of mean BP levels, particularly SBP, in determining future stroke risk in individuals with type 1 diabetes [38]. Furthermore, we failed to demonstrate any prognostic importance of BP-VVV for total CVEs, which included other ‘softer’ events, such as myocardial revascularizations, heart failure, and aortic and peripheral arterial disease events. This is corroborated by a previous study [17], and suggests that these additional macrovascular complications might have different determinants.

### Potential physiopathological mechanisms of increased BP-VVV

The physiopathological mechanisms linking high BP-VVV to cardiovascular morbidity are largely unsettled. Increased BP-VVV has been related to several mechanisms, such as central sympathetic overactivity, variations in renin–angiotensin–aldosterone system stimulation, increased arterial stiffness, and increased production of vasoactive substances; and also to environmental and psychological conditions, including variable adherence to anti-hypertensive treatment [39, 40]. Moreover, there are several methodological factors that may influence the associations between BP-VVV and adverse outcomes, including the number of visits, the time-interval between visits, the BP measurement method (manual or automatic), the duration of follow-up and the level of adjustments for other risk factors [13, 41, 42].

### BP-VVV and microvascular complications in diabetes

For microvascular outcomes, data are more scarce, previous studies were mainly retrospective and most of them investigated the role of BP-VVV as a predictor of renal outcomes [16–19, 21, 25, 26, 43]. Except for the ADVANCE-ON cohort [21], all other studies showed positive associations between increased systolic BP-VVV and abnormal albuminuria development [16, 18, 19, 43] or renal function deterioration [17, 19, 23, 25, 26]. In our study, BP-VVV was a predictor neither of new abnormal albuminuria nor of renal function decline. However, our renal outcomes were different from the previous ones, hindering comparisons with these results. Of note, the renal outcome of the ADVANCE-ON cohort was similar to ours; and, like us, they also did not find prognostic importance of BP-VVV for renal outcomes [21]. Regarding diabetic retinopathy, 3 previous studies [4, 19, 43], like ours, did not find associations between BP-VVV and this outcome; and one study [23] evidenced this association but included cataracts as an outcome. Finally, diabetic peripheral neuropathy development or worsening was also not associated with BP-VVV in the present study, which is in contrast with a unique previous study [23] that reported positive associations between increased systolic BP-VVV and neuropathy. However, in this study the neuropathy outcomes were adjudicated only by ICD coding on administrative database and included, for example, carpal tunnel syndrome [23]; not by directly-examining the presence of diabetic peripheral neuropathy occurrence, as in ours.

### Strengths and limitations

The strengths of the present study are that it is a well-characterized cohort with long-term follow-up, with standardized BP measurements (3–4 per year), and we performed adjustments for serial glycemic and lipid control, physical activity and for the number of anti-hypertensive drugs in use; all factors that could impact on the associations between BP-VVV and outcomes. Further, the endpoints were adjudicated during follow-up by independent observers. However, several limitations also should be noted. First, we could not evaluate the differential effect of various anti-hypertensive drug classes on BP-VVV. Second, the number of events was insufficient to provide precise prognostic information of BP-VVV for some specific outcomes, such as myocardial infarction and stroke. Furthermore, the medium sample size of our cohort may have led to lack of statistical power to show more subtle increases in risk associated with increased BP-VVV. Third, it is a prospective observational cohort; hence no cause-and-effect relationships, nor physiopathological inferences, can be made, but only speculated. Moreover, as with any cohort study, residual confounding due to unmeasured or unknown factors cannot be ruled out. Particularly, BP-VVV is strongly dependent on mean BP levels (the higher mean BPs, the higher the BP-VVV). Although we have adjusted for mean BP levels (Model 3), a residual confounding of increased mean BPs might still have persisted and could, at least partially, explain the significant associations between BP-VVV and the excess risk of MACE outcomes. Finally, it enrolled mainly middle-aged to elderly individuals with long-standing type 2 diabetes followed-up in a tertiary-care university hospital. Hence, our results might not be generalized to younger individuals with recent onset type 2 diabetes or at primary care management.

## Conclusions

This prospective long-term follow-up study showed that systolic BP-VVV was an independent predictor of MACE, but not of total CVEs, cardiovascular and all-cause mortality, and of microvascular complications. Moreover, BP-VVV parameters did not improve overall cardiovascular risk discrimination. Until future interventional studies provide evidences that reduction in BP-VVV may decrease the burden of cardiovascular disease, together with a better standardization of BP-VVV measurements; the main goal of anti-hypertensive treatment in individuals with type 2 diabetes shall remain to be controlling mean BP levels, which has been generally worse than glycemic control [44], not decreasing their visit-to-visit variability.

## Data Availability

The Rio de Janeiro Type 2 Diabetes Cohort Study is an on-going study, and its dataset is not publicly available due to individual privacy of the participants. However, it may be available from the corresponding author on reasonable request.

## Abbreviations

ADVANCE: Action in Diabetes and Vascular Disease, Preterax and Diamicron Modified Release Controlled Evaluation
BP-VVV: Visit-to-visit blood pressure variability
CVE: Cardiovascular event
DBP: Diastolic blood pressure
IDI: Integrated discrimination improvement
MACE: Major adverse cardiovascular events
MI: Myocardial infarction
SBP: Systolic blood pressure
VC: Variation coefficient

## References

[1] Rothwell PM, Howard SC, Dolan E, O’Brien E, Dobson JE, Dahlöf B (2010). Prognostic significance of visit-to-visit variability, maximum systolic blood pressure, and episodic hypertension. Lancet.

[2] Muntner P, Shimbo D, Tonelli M, Reynolds K, Arnett DK, Oparil S (2011). The relationship between visit-to-visit variability in systolic blood pressure and all-cause mortality in the general population: findings from NHANES III, 1988 to 1994. Hypertension.

[3] Hastie CE, Jeemon P, Coleman H, McCallum L, Patel R, Dawson J (2013). Long-term and ultra long-term blood pressure variability during follow-up and mortality in 14,522 patients with hypertension. Hypertension.

[4] Hata J, Arima H, Rothwell PM, Woodward M, Zoungas S, Anderson C, ADVANCE Collaborative Group (2013). Effects of visit-to-visit variability in systolic blood pressure on macrovascular and microvascular complications in patients with type 2 diabetes mellitus: the ADVANCE trial. Circulation..

[5] Mallamaci F, Minutolo R, Leonardis D, D’Arrigo G, Tripepi G, Rapisarda F (2013). Long-term visit-to-visit office blood pressure variability increases the risk of adverse cardiovascular outcomes in patients with chronic kidney disease. Kidney Int.

[6] Lau KK, Wong YK, Chan YH, Teo KC, Chan KH, Wai Li LS (2014). Visit-to-visit blood pressure variability as a prognostic marker in patients with cardiovascular and cerebrovascular diseases-relationships and comparisons with vascular markers of atherosclerosis. Atherosclerosis..

[7] Muntner P, Whittle J, Lynch AI, Colantonio LD, Simpson LM, Einhorn PT (2015). Visit-to-visit variability of blood pressure and coronary heart disease, stroke, heart failure, and mortality: a cohort study. Ann Intern Med.

[8] Wu C, Shlipak MG, Stawski RS, Peralta CA, Psaty BM, Harris TB, Health ABC Study (2017). Visit-to-visit blood pressure variability and mortality and cardiovascular outcomes among older adults: the Health, Aging, and Body Composition study. Am J Hypertens..

[9] Mancia G, Facchetti R, Parati G, Zanchetti A (2012). Visit-to-visit blood pressure variability, carotid atherosclerosis, and cardiovascular events in the European Lacidipine Study on Atherosclerosis. Circulation.

[10] Gao S, Hendrie HC, Wang C, Stump TE, Stewart JC, Kesterson J (2014). Redefined blood pressure variability measure and its association with mortality in elderly primary care patients. Hypertension.

[11] Chang TI, Reboussin DM, Chertow GM, Cheung AK, Cushman WC, Kostis WJ, SPRINT Research Group* (2017). Visit-to-visit office blood pressure variability and cardiovascular outcomes in SPRINT (Systolic Blood Pressure Intervention Trial). Hypertension..

[12] Howard SC, Rothwell PM (2009). Reproducibility of measures of visit-to-visit variability in blood pressure after transient ischaemic attack or minor stroke. Cerebrovasc Dis..

[13] Wang J, Shi X, Ma C, Zheng H, Xiao J, Bian H (2017). Visit-to-visit blood pressure variability is a risk factor for all-cause mortality and cardiovascular disease: a systematic review and meta-analysis. J Hypertens.

[14] Zoppini G, Verlato G, Targher G, Bonora E, Trombetta M, Muggeo M (2008). Variability of body weight, pulse pressure and glycaemia strongly predict total mortality in elderly type 2 diabetic patients. The Verona Diabetes Study. Diabetes Metab Res Rev..

[15] Hsieh YT, Tu ST, Cho TJ, Chang SJ, Chen JF, Hsieh MC (2012). Visit-to-visit variability in blood pressure strongly predicts all-cause mortality in patients with type 2 diabetes: a 5•5-year prospective analysis. Eur J Clin Invest.

[16] Okada H, Fukui M, Tanaka M, Matsumoto S, Mineoka Y, Nakanishi NA (2013). Visit-to-visit blood pressure variability is a novel risk factor for the development and progression of diabetic nephropathy in patients with type 2 diabetes. Diabetes Care..

[17] McMullan CJ, Lambers Heerspink HJ, Parving HH, Dwyer JP, Forman JP, de Zeeuw D (2014). Visit-to-visit variability in blood pressure and kidney and cardiovascular outcomes in patients with type 2 diabetes and nephropathy: a post hoc analysis from the RENAAL study and the Irbesartan Diabetic Nephropathy Trial. Am J Kidney Dis.

[18] Noshad S, Mousavizadeh M, Mozafari M, Nakhjavani M, Esteghamati A (2014). Visit-to-visit blood pressure variability is related to albuminuria variability and progression in patients with type 2 diabetes. J Hum Hypertens.

[19] Takao T, Matsuyama Y, Yanagisawa H, Kikuchi M, Kawazu S (2014). Visit-to-visit variability in systolic blood pressure predicts development and progression of diabetic nephropathy, but not retinopathy, in patients with type 2 diabetes. J Diabetes Complicat.

[20] Takao T, Kimura K, Suka M, Yanagisawa H, Kikuchi M, Kawazu S (2015). Relationships between the risk of cardiovascular disease in type 2 diabetes patients and both visit-to-visit variability and time-to-effect differences in blood pressure. J Diabetes Complicat.

[21] Ohkuma T, Woodward M, Jun M, Muntner P, Hata J, Colagiuri S, ADVANCE Collaborative Group (2017). Prognostic value of variability in systolic blood pressure related to vascular events and premature death in type 2 diabetes mellitus: the ADVANCE-ON study. Hypertension..

[22] Wan EY, Fung CS, Yu EY, Fong DY, Chen JY, Lam CL (2017). Association of visit-to-visit variability of systolic blood pressure with cardiovascular disease and mortality in primary care Chinese patients with type 2 diabetes-a retrospective population-based cohort study. Diabetes Care.

[23] Sohn MW, Epstein N, Huang ES, Huo Z, Emanuele N, Stukenborg G (2017). Visit-to-visit systolic blood pressure variability and microvascular complications among patients with diabetes. J Diabetes Complicat.

[24] Bell KJL, Azizi L, Nilsson PM, Hayen A, Irwig L, Östgren CJ (2018). Prognostic impact of systolic blood pressure variability in people with diabetes. PLoS ONE.

[25] Yu ZB, Wang JB, Li D, Chen XY, Lin HB, Chen K (2019). Prognostic value of visit-to-visit systolic blood pressure variability related to diabetic kidney disease among patients with type 2 diabetes. J Hypertens.

[26] Viazzi F, Bonino B, Mirijello A, AMD-Annals Study Group (2019). Long-term blood pressure variability and development of chronic kidney disease in type 2 diabetes. J Hypertens..

[27] Yu ZB, Li D, Chen XY, Zheng PW, Lin HB, Tang ML, Jin MJ, Wang JB, Chen K (2019). Association of visit-to-visit variability of blood pressure with cardiovascular disease among type 2 diabetes mellitus patients: a cohort study. Diabetes Metab J..

[28] Chiriacò M, Pateras K, Virdis A, Charakida M, Kyriakopoulou D, Nannipieri M (2019). Association between blood pressure variability, cardiovascular disease and mortality in type 2 diabetes: a systematic review and meta-analysis. Diabetes Obes Metab.

[29] Salles GF, Leite NC, Pereira BB, Nascimento EM, Cardoso CR (2013). Prognostic impact of clinic and ambulatory blood pressure components in high-risk type 2 diabetic patients: the Rio de Janeiro Type 2 Diabetes Cohort Study. J Hypertens.

[30] Cardoso CR, Leite NC, Ferreira MT, Salles GF (2015). Prognostic importance of baseline and serial glycated hemoglobin levels in high-risk patients with type 2 diabetes: the Rio de Janeiro Type 2 Diabetes Cohort Study. Acta Diabetol.

[31] Cardoso CR, Moran CB, Marinho FS, Ferreira MT, Salles GF (2015). Increased aortic stiffness predicts future development and progression of peripheral neuropathy in patients with type 2 diabetes: the Rio de Janeiro Type 2 Diabetes Cohort Study. Diabetologia.

[32] Cardoso CRL, Leite NC, Dib E, Salles GF (2017). Predictors of development and progression of retinopathy in patients with type 2 diabetes: importance of blood pressure parameters. Sci Rep..

[33] Cardoso CRL, Leite NC, Salles GC, Ferreira MT, Salles GF (2018). Aortic stiffness and ambulatory blood pressure as predictors of diabetic kidney disease: a competing risks analysis from the Rio de Janeiro Type 2 Diabetes Cohort Study. Diabetologia.

[34] Santos TRM, Melo JV, Leite NC, Salles GF, Cardoso CRL (2018). Usefulness of the vibration perception thresholds measurement as a diagnostic method for diabetic peripheral neuropathy: results from the Rio de Janeiro type 2 diabetes cohort study. J Diabetes Complicat.

[35] DeLong ER, DeLong DM, Clarke-Pearson DL (1988). Comparing the areas under two or more correlated receiver operating characteristic curves: a nonparametric approach. Biometrics.

[36] Pencina MJ, D’Agostino RB, D’Agostino RB, Vasan RS (2008). Evaluating the added predictive ability of a new marker: from area under the ROC curve to reclassification and beyond. Stat Med.

[37] Pencina MJ, D’Agostino RB, Demler OV (2012). Novel metrics for evaluating improvement in discrimination: net reclassification and integrated discrimination improvement for normal variables and nested models. Stat Med.

[38] Hägg-Holmberg S, Dahlström EH, Forsblom CM, Harjutsalo V, Liebkind R, Putaala J, Tatlisumak T, Groop PH, Thorn LM, FinnDiane Study Group (2019). The role of blood pressure in risk of ischemic and hemorrhagic stroke in type 1 diabetes. Cardiovasc Diabetol..

[39] Parati G, Ochoa JE, Salvi P, Lombardi C, Bilo G (2013). Prognostic value of blood pressure variability and average blood pressure levels in patients with hypertension and diabetes. Diabetes Care.

[40] Parati G, Ochoa JE, Lombardi C, Bilo G (2015). Blood pressure variability: assessment, predictive value, and potential as a therapeutic target. Curr Hypertens Rep.

[41] Stevens SL, Wood S, Koshiaris C, Law K, Glasziou P, Stevens RJ (2016). Blood pressure variability and cardiovascular disease: systematic review and meta-analysis. BMJ..

[42] Veloudi P, Sharman JE (2018). Methodological factors affecting quantification of blood pressure variability: a scoping review. J Hypertens.

[43] Takao T, Suka M, Yanagisawa H, Matsuyama Y, Iwamoto Y (2017). Predictive ability of visit-to-visit variability in HbA1c and systolic blood pressure for the development of microalbuminuria and retinopathy in people with type 2 diabetes. Diabetes Res Clin Pract.

[44] McAlister FA, Lethebe BC, Lambe C, Williamson T, Lowerison M (2018). Control of glycemia and blood pressure in British adults with diabetes mellitus and subsequent therapy choices: a comparison across health states. Cardiovasc Diabetol..

